# *Rhipicephalus sanguineus* Complex in the Americas: Systematic, Genetic Diversity, and Geographic Insights

**DOI:** 10.3390/pathogens10091118

**Published:** 2021-09-01

**Authors:** Sokani Sánchez-Montes, Beatriz Salceda-Sánchez, Sergio E. Bermúdez, Gabriela Aguilar-Tipacamú, Gerardo G. Ballados-González, Herón Huerta, Mariel Aguilar-Domínguez, Jesús Delgado-de la Mora, Jesús D. Licona-Enríquez, David Delgado-de la Mora, Andrés M. López-Pérez, Marco A. Torres-Castro, Virginia Alcántara-Rodríguez, Ingeborg Becker, Pablo Colunga-Salas

**Affiliations:** 1 Facultad de Ciencias Biológicas y Agropecuarias, Región Tuxpan, Universidad Veracruzana, Tuxpan de Rodríguez Cano, Veracruz 92870, Mexico; sok10108@gmail.com; 2 Centro de Medicina Tropical, Unidad de Investigación en Medicina Experimental, Facultad de Medicina, Universidad Nacional Autónoma de México, Mexico City 04510, Mexico; becker@servidor.unam.mx; 3 Laboratorio de Entomología, Instituto de Diagnóstico y Referencia Epidemiológicos, Secretaría de Salud, Mexico City 01480, Mexico; cerato_2000@yahoo.com; 4 Departamento de Investigación en Entomología Médica, Instituto Conmemorativo Gorgas de Estudios de la Salud, Panama 0816-02593, Panama; sbermudez@gorgas.gob.pa; 5 C. A. Salud Animal y Microbiología Ambiental, Facultad de Ciencias Naturales, Universidad Autónoma de Querétaro, Querétaro 76750, Mexico; gabriela.aguilar@uaq.mx; 6 Facultad de Medicina Veterinaria y Zootecnia, Universidad Veracruzana, Veracruz 91710, Mexico; balladosgerardo506@hotmail.com (G.G.B.-G.); marieaguilar@uv.mx (M.A.-D.); 7 Departamento de Anatomía Patológica, Instituto Nacional de Ciencias Médicas y Nutrición Salvador Zubirán, Mexico City 14080, Mexico; jdelgadom1992@gmail.com; 8 Unidad Médica de Alta Especialidad Hospital de Pediatría, Centro Médico Nacional Siglo XXI, Mexico City 06720, Mexico; jdliconae@gmail.com; 9 Departamento de Patología, Facultad de Medicina Veterinaria y Zootecnia, Universidad Nacional Autónoma de México, Mexico City 04510, Mexico; ddm070796@gmail.com; 10 School of Veterinary Medicine, Department of Medicine and Epidemiology, University of California, Davis, CA 95616, USA; amlope@ucdavis.edu; 11 Laboratorio de Ecología de Enfermedades y Una Salud, Departamento de Etología, Fauna Silvestre y Animales de Laboratorio, Facultad de Medicina Veterinaria y Zootecnia, Universidad Nacional Autónoma de México, Mexico City 04510, Mexico; 12 Laboratorio de Enfermedades Emergentes y Reemergentes, Centro de Investigaciones Regionales “Dr. Hideyo Noguchi”, Universidad Autónoma de Yucatán, Yucatán 97000, Mexico; antonio.torres@correo.uady.mx; 13 Unidad Departamental de Vigilancia Epidemiológica, Secretaría de Salud de la Ciudad de México, Mexico City 06900, Mexico; vealcant55@yahoo.com; 14 Instituto de Biotecnología y Ecología Aplicada, Universidad Veracruzana, Xalapa de Enríquez, Veracruz 91090, Mexico

**Keywords:** brown dog tick, vector, genetic diversity, Tropical linage

## Abstract

The *Rhipicephalus sanguineus* group encompasses at least 12 validated species of Palearctic and Afrotropical hard ticks, which are relevant in veterinary medicine and public health. The taxonomy of *R. sanguineus* s.s., has been particularly intensely debated, due to its wide geographic distribution, morphological variants, parasite-host associations, and its capacity and vectorial competence for the transmission of several pathogens. By sequencing mitochondrial markers, it was possible to identify the existence of multiple lineages, among which the Tropical and the Temperate lineages stand out, particularly in America. However, the northern limit between these lineages is not clear due to the lack of extensive sampling across Mexico. For this reason, the aim of the present study was to determine the genetic diversity and structure of the *R. sanguineus* group in Mexico and to compare it with the populations reported in the Americas, in order to propose the northern limit of the *R. sanguineus* Tropical lineage and the potential regions of sympatry with *R. sanguineus* s.s. The findings of this study now confirm the presence of *R. sanguineus* s.s. in Mexico, showing a subtle genetic structure and high genetic diversity throughout its distribution in the Americas. In contrast, the Tropical lineage seems to be genetically less diverse in its overall distribution in the Americas. The genetic diversity of these two independent lineages could have important epidemiological implications in the transmission of tick pathogens.

## 1. Introduction

The *Rhipicephalus sanguineus* group encompasses at least 12 validated species (*R. camicasi*, *R. guilhoni*, *R. leporis*, *R. moucheti*, *R. pumilio*, *R. pusillus*, *R. rossicus*, *R. schulzei*, *R. sulcatus*, *R. turanicus*, *R. sanguineus*, and the recently described *R. afranicus*) of Palearctic and/or Afrotropical hard ticks widely distributed worldwide by several historical colonization processes, which exhibit a high relevance in veterinary medicine and public health due to direct damage caused by infestation in the hosts and by the transmission of pathogens [1,2]. *Rhipicephalus sanguineus* s.s., also known as the brown dog tick, is a species first obtained from canid hosts in Gallia (now France) in 1806 by Latreille [3]. However, systematised studies of ticks infesting canid carried out worldwide have marked morphological differences between the populations of Northwest Africa and those introduced in Australia and America [4,5]. These differences were added to other taxonomic problems, derived from the lack of voucher specimens and the poor morphological description of *R. sanguineus* [6].

The morphological variations included differences in sizes and shapes of the spiracular plate, as well as in the shape and arrangement of the adanal plates, in addition to particular parasite-host associations, which increased the controversy over the existence of a single species. For this reason, new tools, such as molecular biology, were added to elucidate the status of this species. By sequencing mitochondrial markers, it was possible to identify the existence of multiple lineages, among which the Tropical (*R. sanguineus* s.l.) and the Temperate lineages (*R. sanguineus* s.s.) stand out [4,7,8]. Consequently, new taxonomic works were developed to obtain a neotype, with the morphological description of all its stages (larva, nymph, and adult), and the generation of mitochondrial and nuclear sequences that would make it possible to untangle the taxonomic problem exhibited by the *R*. *sanguineus* group complex [6].

The Temperate and Tropical lineages have been reported across the Americas [4,5,6,8,9,10,11]. It has been proposed that the Tropical lineage occurs between the Tropics of Capricorn and Cancer, whereas the Temperate lineage was expected to be found above or below the Tropics [5,9,11]. However, the northern limit between these lineages is not clear due to the lack of an extensive sampling across Mexico, since only sequences from specimens collected near the US-Mexico border have been used [5,11]. Considering the biogeographic regionalisation proposed by Morrone et al. [12], and that the limit between the Nearctic and Neotropical regions occurs in Mexico, we formulated the hypothesis that the northern limits of both lineages of the *R*. *sanguineus* group must be in accordance with this biogeographic regionalisation.

Several populations of both linages are well-known vectors of tick-borne pathogens such as *Rickettsia rickettsii* and *Ehrlichia canis*, which cause Rocky Mountain Spotted Fever and canine monocytic ehrlichiosis in America, respectively [9,13,14,15,16]. Surveillance and experimental studies have suggested that each lineage exhibits a marked vectorial capacity, associated with the transition of a particular group of microorganisms. Thus, *R. sanguineus* s.s. (formerly known as the Temperate lineage) is capable of transmitting *Anaplasma platys*, and *Rickettsia massiliae* to dogs and humans, respectively, in Argentina and the US [17,18,19,20,21]. Additionally, *R. sanguineus* s.s. has been experimentally confirmed as a competent vector of *A. platys* in the rabbit model [20]. On the other hand, the Tropical lineage is considered the main vector of *E. canis* in the US, Mexico, and the Caribbean [22]; moreover, *A. platys* has been reported in regions of Central America, coincidentally with the Tropical lineage [23]. However, no tests have been carried out to verify the vectorial capacity of this linage. Otherwise, *R*. *sanguineus* s.s. can be naturally infected by *E*. *canis* in Argentina [24,25]. For this reason, in regions where both lineages are sympatrically distributed, it is possible to assume that a high richness of tick-borne pathogens could be circulating.

In Mexico, a few studies have been carried out to monitor the circulating lineages of *R. sanguineus* s.l. Studies based on amplification of the mitochondrial gene *16S rDNA* have permitted to detect the Tropical lineage in specimens from Baja California, Chihuahua, Guanajuato, and Oaxaca [26,27,28]. Furthermore, the presence of *R. sanguineus* s.s. had been suspected due to the detection of *R. massiliae* and *A. platys* in ticks and dogs in the border states of Baja California, Chihuahua, and Durango [27,29,30,31].

Yet, due to its important and variable role in the transmission of several tick-borne pathogens, it is crucial to obtain more robust information on the identity and diversity of the *R. sanguineus* group circulating in the Americas, particularly in Mexico, where two zoogeographical regions converge. In this context, we evaluated the genetic diversity and structure of the *R*. *sanguineus* group in Mexico and compared it with the populations reported in the Americas, with the aim to propose the northern limit of the *R*. *sanguineus* Tropical Lineage and the potential regions of sympatry with *R*. *sanguineus* s.s.

## 2. Results

### 2.1. Molecular Identification

**Figure 1:**
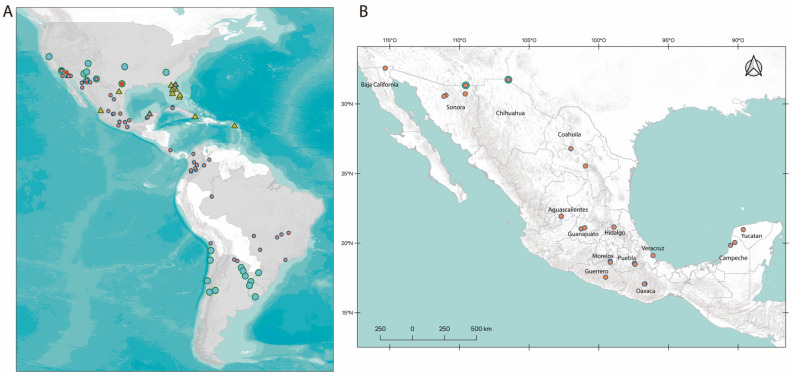
Distribution of *Rhipicephalus sanguineus* s.s. and *R*. *sanguineus* s.l. in the Americas. (**A**) Records of *R*. *sanguineus* s.l. in the Americas based on previously reports; shaded countries have at least one record. (**B**) Sampling done in this study throughout Mexico. Blue circles correspond to *R*. *sanguineus* s.s. and orange circles to *R*. *sanguineus* s.l. Tropical Lineage based on records of the *16S rDNA* gene. Red hexagons correspond to *R. sanguineus* s.s. records with records from the literature using the *12S-rDNA* gene sequences available in GenBank; similarly, yellow triangles correspond to *R*. *sanguineus* s.l. Tropical Lineage. A layer from ESRI Terrain was used to construct the figure (https://server.arcgisonline.com/ArcGIS/rest/services/World_Terrain_Base/MapServer/tile/{z}/{y}/{x}, accessed on 30 August 2021). A detailed list of localities is available in Supplementary File 1.

A total of 52 ticks from 23 localities in 14 of the 32 Mexican states were selected for the analysis (Figure 1B; Table 1). 

From all specimens, partial sequences of mitochondrial *16S rDNA* gene were obtained and included in the phylogenetic approaches and the genetic analyses. In the final alignment of 372 base pairs (including outgroups), 299 conserved sites (80.3%), 32 parsimony informative sites (8.6%), and 72 variable sites (19.3%) were detected. However, considering only the *R*. *sanguineus* s.l. or Tropical Lineage across its entire distribution in the Americas, 362 conserved sites (97.3%), 4 parsimony informative sites (1%) and 9 variable sites (2.4%) were obtained. On the other hand, for the *R*. *sanguineus* s.s., 362 conserved sites (97.3%), 6 parsimony informative sites (1.6%), and 7 variable sites (1.8%) were identified.

Further examination of the obtained DNA sequences revealed two main groups: the first group was formed by our isolates CMT-02 (MZ618799) and CMT-30 (MZ618826), which were 100% identical to each other and 99.73% identical to the *R. sanguineus* isolate FRA3 (MH630344) sequence generated in the description of the neotype of *R. sanguineus* by Nava et al. [6], and 99.73% were identical to the Piacenza-A (KX793736) sequence. On the other hand, the remaining 50 isolates were 100% identical to each other and to other isolates from Mexico (MK680295), Brazil (MF351603), and Colombia (MF351598).

### 2.2. Phylogenetic Analysis

**Figure 2:**
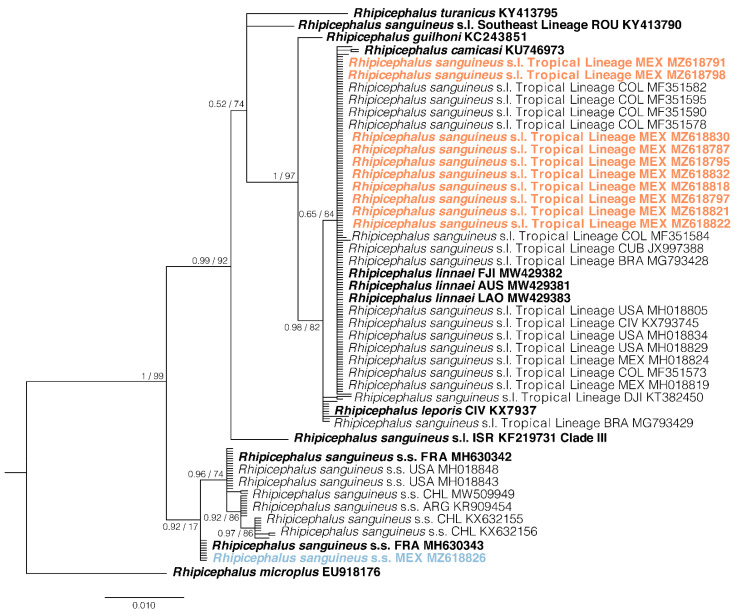
Bayesian Inference phylogenetic compress analysis of the *16S rDNA* partial gene for *Rhipicephalus sanguineus* s.s. and *Rhipicephalus sanguineus* s.l. Tropical lineage from the Nearctic and Neotropical regions, as well as of a few selected genes from the Afrotropical, Australasian, and Oriental regions. The phylogenetic relationships were inferred based on the Hasegawa-Kishino-Yano (HKY85) with gamma distribution for a total of 372 bp. Branch labels show branch support at each node according to posterior probabilities/non-parametric bootstrap, from the BI and ML analyses, respectively. Orange and blue sequences correspond to sequences obtained in this study.

The best substitution models were the HKY + F + G4 for the ML analysis (BIC = 2636.2415) and the HKY + G for the BI analysis (BIC = 4424.872). In both analyses, the same topology was recovered; however, in general, the branch support was stronger in the BI results (Figure 2). 

Both phylogenetic inferences revealed two well-defined and supported monophyletic groups. One group formed by all sequences of *R. sanguineus* s.s., with phylogenetic relationships well resolved. On the other hand, within the *R. sanguineus* group, the phylogenetic associations were not completely resolved, since only Clade III was resolved, but the relationships among the groups formed by *R*. *turanicus*, *R*. *guilhoni*, *R*. *camicasi, R*. *leporis*, and the Tropical Lineage were not resolved (Supplementary File 2).

### 2.3. Genetic Analysis

**Figure 3:**
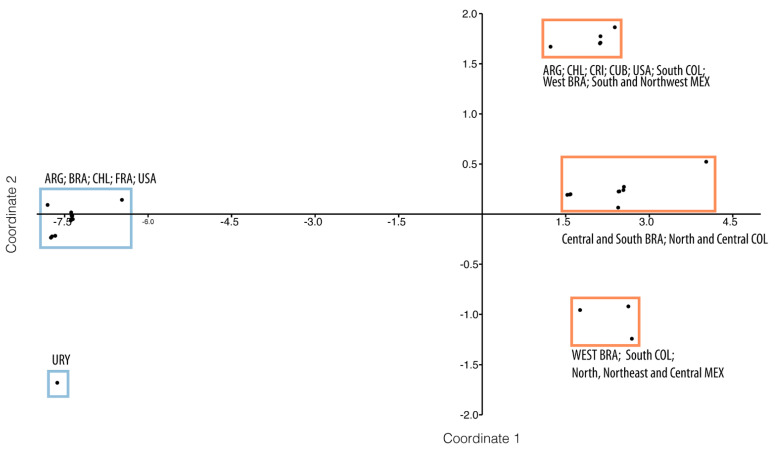
PCoA plot of the first and second coordinates based on the eigenvalue scale and Euclidean distances for the 191 sequences of *Rhipicephalus sanguineus* and *Rhipicephalus sanguineus* s.l. Tropical Lineage from the Americas used in this study. The proportion of total variance along the two coordinates were 84.094% and 7.0986%, the eigenvalues were 3435.9 and 290.04, respectively. Orange sequences correspond to *R*. *sanguineus* s.l. Tropical Lineage, and blue sequences to the *R*. *sanguineus*. We used the ISO 3166-1 alfa-3 code for the countries.

According to the phylogenetic analysis, the PCoA also revealed two well-distinctive molecular groups distributed in the Americas: the *R. sanguineus* s.s. and the *R. sanguineus* s.l. Tropical Lineage. In the specific case of *R. sanguineus* s.s., only two well-defined subpopulations were evidenced, with the Uruguay isolate (GU553084) being the only one differing from the remaining 45 sequences from Argentina, Brazil, Chile, and the US (Figure 3). 

In the case of the Tropical Lineage, three subpopulations were found. The first one was formed by sequences from Argentina, Chile, Costa Rica, Cuba, the US, the South of Colombia, the West of Brazil, and the South and Northwest of Mexico. The second subpopulation was formed by sequences from Central and South Brazil and both North and Central Colombia. The last subpopulation was formed by sequences from West Brazil, South Colombia, and North, Northeast and Central Mexico (Figure 3).

**Figure 4:**
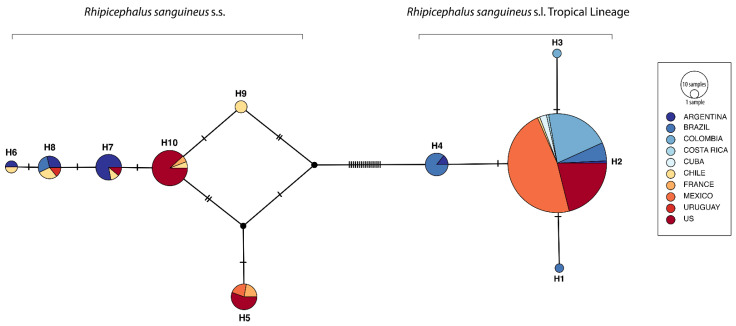
Haplotype network for the *Rhipicephalus sanguineus* s.l. Tropical Lineage and *Rhipicephalus sanguineus* s.s. from the Americas. The colours correspond to the country of origin of each haplotype. Black lines represent the mutational steps between each haplotype. Black dots represent a putative haplotype that must exist to link the network.

A total of 10 haplotypes were detected in the sequences from the Americas, with a haplotype diversity (Hd) of 0.4863 and a nucleotide diversity (π) of 0.0258. However, two groups could also be defined in the haplotype network, since 16 mutations divided the haplotypes into two groups: H1–H4 corresponded to the Tropical lineage, and the second group, formed by haplotypes H5–H10, corresponded to *R*. *sanguineus* s.s. Even though these two groups were well-supported, the H2 haplotype was the most abundant one of the networks, including samples from 8 of the 10 considered countries (80%), namely Argentina, Brazil, Chile, Colombia, Costa Rica, Cuba, Mexico, and the US. On the other hand, for the second group of haplotypes, no differentiation centre could be defined (Figure 4).

**Figure 5:**
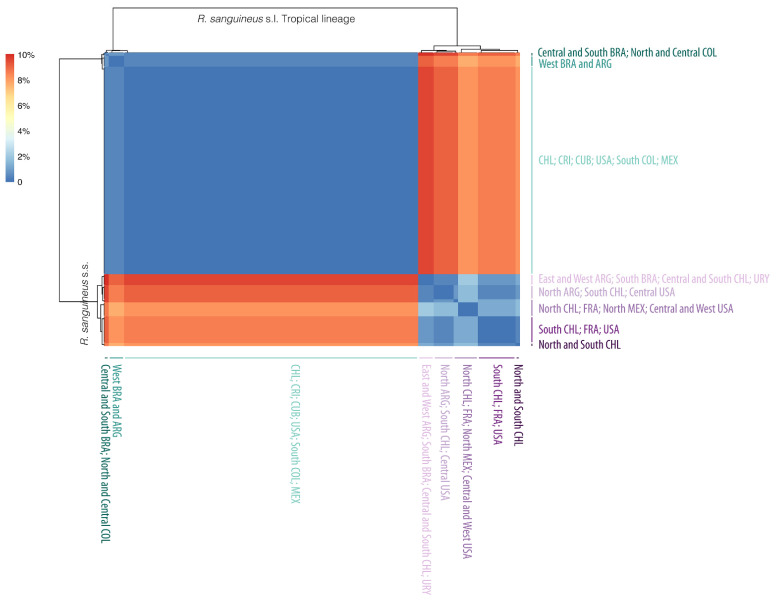
Heatmap of Nei’s genetic distances for *Rhipicephalus sanguineus* s.s. and *Rhipicephalus sanguineus* s.l. Tropical lineage from the Americas. Colour gradient represents the percentage of differentiation when comparing the 191 sequences according to the scale bar in the right.

Finally, with the Nei’s distances, the group of *R. sanguineus* s.l. Tropical Lineage and the *R. sanguineus* s.s. were well differentiated by a difference of 7.7–10%. Only *R. sanguineus* s.s. revealed some genetic structure; however, the higher pairwise distances within this group were 2.1% between two groups: one was formed by the two sequences of this group from Mexico (isolates CMT-02 [MZ618799] and CMT-30 [MZ618826]), four sequences from the US (KT382465, MH018810, MH018811, MH018813), and two from France (MH630343 and MH630344). The second group was formed by two sequences from Brazil (MF477856 and KX533942), Argentina (KU498302 and MT152825), and Chile (MW509950 and KX632155), as well as the single sequence from Uruguay (GU553084) [Figure 5; Supplementary File 3].

### 2.4. Geographic Distribution

We recovered a total of 202 geo-referred occurrence records for the *R. sanguineus* group from the Americas. A total of 156 (77.3%) corresponded to the Tropical lineage and the remaining 46 records (22.7%) were from *R. sanguineus* s.s. The Tropical lineage is widely distributed across the Neotropical Region; meanwhile, *R. sanguineus* s.s. is restricted to North America, including the US and the US-Mexico border, and is also present in the South American Cone, where it is restricted to Argentina, Chile, and Uruguay (Figure 1; Supplementary File 4).

The northwester limit of the Tropical Lineage is in Lytle Creek, CA, USA, while the Northeastern limit is in St. Johns, FL, USA, with two additional Northwestern records in San Diego and Imperial, CA, USA, and one Northeaster record in Gilchrist, FL, USA. On the other hand, their Southwestern limit is in the department of Matacos, Formosa, ARG, and their Southeastern limit is in the municipality of Saõ Paulo, Saõ Paulo, BRA, with one more Southwestern locality in the department of Rivadavia, Escalante, ARG. The Northern limit of these two lineages includes three sympatric localities, one in Lytle Creek, CA, USA, and two Mexican border cities: Ciudad Juárez, Chihuahua, and Agua Prieta, Sonora (Figure 1). The QGIS project with the shapefiles and coordinates is available: 10.5281/zenodo.4739562.

## 3. Discussion

This study represents the first effort to address the Northern limit of the Temperate and Tropical lineages of the *R*. *sanguineus* group in the Americas. Our findings are concordant with those reported by Nava et al. [6], assigning the previous known Temperate lineage as *R*. *sanguineus* s.s., since in our phylogenetic analyses, the sequences from the neotype of *R*. *sanguineus* s.s. were grouped in a single monophyletic clade with the sequences previously assigned to the Temperate lineage [5,11].

### 3.1. Genetic Diversity

Genetically, both lineages were highly supported, as has been reported in previous phylogenetic studies [4,5,6,8,10,11]. In general, the genetic information obtained from the analysed fragment of the *16S rDNA* gene was sufficient and robust to delimitate both lineages; however, the genetic diversity was low, with an Hd of 0.4863 and π value of 0.0258.

Specifically, this gene fragment revealed subtle genetic differentiation across the distribution of both lineages. Regarding *R*. *sanguineus* s.s., two main subpopulations are differentiated, with one being the subpopulation formed by the single sequence from Uruguay. In contrast, the *R*. *sanguineus* Tropical Lineage is at least formed by three subpopulations with no apparent geographical correlation. However, further genetic structure studies must be done using other genetic markers, such as microsatellites or genes with a higher mutation rate that could provide more recent information about their genetic diversity, such as the cytochrome oxidase subunit 1 (*COI*), the cytochrome b (*cytB*), or the second internal transcribed spacer of the nuclear ribosomal gene cluster (*ITS2*) to further characterise the subpopulations [32,33,34].

Although the numbers of available sequences from *R*. *sanguineus* s.s. were lower than those from the Tropical Lineage, when these two lineages were compared with the haplotype network, *R*. *sanguineus* s.s. seems to be more diverse than the Tropical lineage, with six haplotypes of the total 10. This was also supported by the genetic distances, where a subtle genetic structure could be shown within this species in North and South America. This obvious genetic diversity could be due to an isolation-by-distance effect or by two independent colonization events, that may have been influenced by multiple factors related to radiation across several new hosts and climate niche partitioning, as was recently proposed by Bakkes et al. [2]. However, these hypotheses must be addressed in further studies.

Contrary to the information obtained with *R*. *sanguineus* s.s., the use of the *16S rDNA* gene fragment seems to be less informative for the Tropical lineage, containing only four haplotypes and without an obvious genetic structuring. However, it is important to note that in this case, a single haplotype (H2) concentrates most of the diversity and could be inferred as the differentiation centre, since all mentioned countries are included in this haplotype. This has been proposed for other biological systems, with some unique haplotypes from South America (Colombia and Brazil) as tip haplotypes [35,36,37]. This phenomenon could be explained by a long-distance founding event, based on the great vagility of the hosts, such as in several populations of *Amblyomma maculatum* within the US [38].

This differentiation might be epidemiologically relevant due to variability in the vectorial capacity and the competence of some of the populations of the *R*. *sanguineus* group to transmit, for example, *E*. *canis* in Brazil [9].

### 3.2. Geographic Distribution

Based on the biogeographic regionalisation, no correlation could be evidenced between the presence of *R*. *sanguineus* s.s. and that of the Tropical lineage. In the Northern limit, the two sympatric locations for both lineages are highly crowded border crossings that allow a constant exchange between the populations of *R. sanguineus* of the US and Mexico through the movement of domestic animals.

On the other hand, in South America, where a more complex biogeographic regionalisation is found, due to the complexity of the biota and fauna, *R*. *sanguineus* s.s. and Tropical linage only share the Chacoan province in Argentina. In this context, the use of the distribution proposed in this study must be applied with caution until future efforts allow us to complete the distribution analysis in this area.

To conclude, the findings of this study confirm that *R*. *sanguineus* s.s. corresponds to the previously known “Temperate Lineage”, containing a subtle genetic structure and high genetic diversity throughout its distribution in the Americas. In contrast, the *16S rDNA* region seems to be less informative for inferring the genetic diversity of the Tropical lineage in its overall distribution in the Americas. Yet caution is warranted, since the analysis of the *16S rDNA* gene fragment does not permit us to untangle the phylogenetic taxonomy of this complex group formed by other Palearctic and/or Afrotropical species, such as *R*. *turanicus*, *R*. *sanguineus* Southeast Lineage, *R*. *guilhoni*, *R*. *leporis*, and *R*. *camicasi*. 

We also now propose the first fine scale map with the boundaries of these two lineages of the *R. sanguineus* group in the Americas, showing different biogeographical distributions. Finally, the genetic diversity shown by these two independent lineages could have important epidemiological implications in the transmission of tick pathogens.

## 4. Materials and Methods

### 4.1. Tick Collection and Identification

As a part of the ‘National Program for *Rickettsia* Surveillance’, we received brown dog ticks (*R. sanguineus* group) from the National Network of Public Health Laboratories of the Mexican states of Aguascalientes, Baja California, Campeche, Chihuahua, Coahuila, Guanajuato, Guerrero, Hidalgo, Morelos, Oaxaca, Puebla, Sonora, Veracruz, and Yucatan. Additionally, we analysed several ticks from previous tick-borne surveillance studies carried out in Chihuahua, Sonora, and Veracruz [27,39,40]. With this extensive sampling, we ensured the representation of both biogeographic regions according to Morrone [12]: the Nearctic Region (Baja California, Chihuahua, Coahuila, Sonora), and the Neotropical Region (Campeche, Guanajuato, Hidalgo, Puebla, Oaxaca, Veracruz, and Yucatan).

All ticks were fixed and preserved in 70% ethanol and morphologically identified with an Olympus SZX7 stereoscopic microscope, using the specialised taxonomic keys of Keirans and Litwak [41], Dirección General de Salud Animal [42], de Oliveira et al. [43], and Nava et al. [6]. Each specimen was transferred to a 1.5-mL Eppendorf^®^ tube and cut into small pieces using a sterile scalpel blade for each specimen to avoid cross contamination between tick samples. The samples were then macerated with a 1.5-pellet pestle.

The DNA extraction was carried out using the Qiagen “DNeasy Blood & Tissue Kit”^®^, implementing the “Purification of Total DNA from Animal Tissues (Spin-Column Protocol)”. For molecular identification of the tick species, a segment of approximately 400 bp of the mitochondrial *16S rDNA* gene was amplified in all samples, using the primers 16S + 1/16S − 1 and the cycling conditions reported by Norris et al. [44]. The reaction mixture consisted of 12.5 μL GoTaq^®^ Green Master Mix, 2X Promega Corporation (Madison, WI, USA), the pair of primers (100 ng each), 6.5 μL nuclease-free water, and 30 ng DNA in a final volume of 25 μL. Negative (reaction mix without the DNA template) and positive (*Amblyomma ovale* DNA; GenBank Accession number MW386402) controls were included. The PCR products were resolved in 2% agarose gels using SmartGlow™ Pre-Stain of Accuris Instruments^®^ (Edison, NJ, USA) and visualised by UV-transillumination. Purified amplification products were submitted for sequencing at Macrogen Inc., Seoul, Korea.

### 4.2. Phylogenetic Analysis

Recovered sequences were edited using FinchTV 1.5 (Geospiza, Inc., Seattle, WA, USA), conducting a visual inspection. The final sequences were compared to each other to identify inter/intra species variation, using the BLASTn tool from NCBI (https://blast.ncbi.nlm.nih.gov/Blast.cgi?PROGRAM=blastn&PAGE_TYPE=BlastSearch&LINK_LOC=blasthome). 

We then performed multiple alignments according to the secondary structure of the *16S rDNA* structure, using the Q-INS-i algorithm in the MAFFT version. The set of sequences included all available sequences of *R*. *sanguineus* s.l. from the Americas in GenBank, with the sequences of the neotype of *R*. *sanguineus* from France. Following the recommendations of Guglielmone et al. [1], we included sequences of other species of the *Rhipicephalus sanguineus* group, *R*. *turanicus*, *R*. *guilhoni*, *R*. *leporis*, *R*. *camicasi*, and *R*. *sanguineus* tropical lineage from the Australasian, Oriental, and Afrotropical regions according to Chitimia–Dobler et al. [45], Hornok et al. [46], and Slapeta et al. [47]. Additionally, we also included sequences of the south-eastern Lineage, *R. sanguineus* s.l. clade III [according to Nava et al. [6] and *Rhipicephalus microplus*, as an outgroup. The complete list of these sequences and their geographical distribution are presented in Supplementary File 1. Published geographical localities were referenced using the electronic gazetteer “Global Gazeteer V. 2.3”.

Two phylogenetic analyses were performed, and the best substitution model was evaluated differentially, following the recommendations by Colunga-Salas and Hernández–Canchola [48]. The first phylogenetic approach was evaluated in IQ-TREE [49], and the best-fit substitution model was assessed with the ModelFinder algorithm [50], considering a full-tree search for each model and the Bayesian information criterion (BIC). The Maximum Likelihood (ML) hypothesis was estimated using the substitution model that was previously calculated, with 10,000 replicates of non-parametric bootstrap to evaluate branch support.

The second approach was performed using MrBayes 3.2 [51]. To calculate the best-fit substitution model, PartitionFinder 2 with the *mrbayes* models and the *greedy* scheme search [52,53] were assessed while also considering the BIC. The Bayesian inference (BI) hypothesis was estimated using the Markov Chain Monte Carlo (MCMC) algorithm and the substitution model that was previously calculated. For this, three hot and one cold chain in two independent runs of 20 million generations, sampling every 1000 generations, were performed. The final topology was obtained using a majority consensus tree, considering a burn-in of 30%. The convergence of results and good sampling (ESS > 200) were evaluated in Tracer 1.7.1 [54].

### 4.3. Genetic Analysis

For all following analyses, we only used the multiple alignment of the *R*. *sanguineus* Tropical Lineage and *R*. *sanguineus* s.s. from the Nearctic and Neotropical regions, without the outgroup. To provide a more detailed account of the genetic diversity and to detect possible populations and subpopulations in the *R*. *sanguineus* Tropical Lineage and *R*. *sanguineus* s.s., an ordination PCoA of the *eigenvalue* scores was performed in PAST 4.04 [55], based on the Euclidean distances [37].

Haplotype networks with mutational step estimations were constructed using the median-joining network in POPART 1.7 [56]. Additionally, Nei’s genetic distances were calculated between the sequences in the *adegenet* R package [57], considering the substitution models previously calculated for the multiple alignment.

## Figures and Tables

**Table 1:** Collection sites of *R*. *sanguineus* group in Mexico.

GenBank Accession Number	Species	Sex	Source	Collection Date	State	Municipality	Locality	Longitude	Latitude
MZ618782	Tropical Linage	♀	Dog	11 September 2019	Aguascalientes	Calvillo	El Mirador	−102.694	21.933
MZ618783	Tropical Linage	♀	Dog	11 September 2019	Aguascalientes	Calvillo	El Mirador	−102.694	21.933
MZ618784	Tropical Linage	♀	Dog	11 September 2019	Aguascalientes	Calvillo	El Mirador	−102.694	21.933
MZ618785	Tropical Linage	♀	Dog	19 September 2019	Baja California	Mexicali	Ejido Sinaloa	−115.327	32.572
MZ618786	Tropical Linage	♀	Dog	19 September 2019	Baja California	Mexicali	Ejido Sinaloa	−115.327	32.572
MZ618787	Tropical Linage	♀	Dog	19 September 2019	Baja California	Mexicali	Alianza para la Producción (La Choyera)	−115.327	32.572
MZ618788	Tropical Linage	♀	Dog	9 September 2019	Baja California	Mexicali	Ejido Cuernavaca	−115.327	32.572
MZ618789	Tropical Linage	♀	Dog	9 September 2019	Baja California	Mexicali	Ejido Cuernavaca	−115.327	32.572
MZ618790	Tropical Linage	♂	Environment	24 June 2019	Campeche	Tenabo	Tenabo	−90.225	20.040
MZ618791	Tropical Linage	♂	Environment	24 June 2019	Campeche	Tenabo	Tenabo	−90.225	20.040
MZ618792	Tropical Linage	♂	Environment	24 June 2019	Campeche	Campeche	Campeche	−90.532	19.842
MZ618793	Tropical Linage	♀	Environment	20 June 2019	Coahuila	Cuatrociénegas de Carranza	Antiguos Mineros del Norte (Santa Tecla)	−102.001	26.783
MZ618794	Tropical Linage	♀	Environment	20 June 2019	Coahuila	Cuatrociénegas de Carranza	Antiguos Mineros del Norte (Santa Tecla)	−102.001	26.783
MZ618795	Tropical Linage	♀	Dog	20 June 2019	Coahuila	Cuatrociénegas de Carranza	Antiguos Mineros del Norte (Santa Tecla)	−102.001	26.783
MZ618796	Tropical Linage	♂	Dog	8 July 2019	Coahuila	Cuatrociénegas de Carranza	Cuatrociénegas de Carranza	−102.067	26.986
MZ618797	Tropical Linage	♂	Environment	25 June 2019	Coahuila	Ramos Arispe	Cerro Cruz	−100.951	25.541
MZ618798	Tropical Linage	♀	Dog	27 June 2019	Chihuahua	Juárez	Juárez	−106.487	31.739
MZ618799	*R. sanguineus* s.s.	♀	Dog	4 July 2019	Chihuahua	Juárez	Juárez	−106.487	31.739
MZ618800	Tropical Linage	♀	Dog	8 July 2019	Chihuahua	Juárez	Juárez	−106.487	31.739
MZ618801	Tropical Linage	♀	Environment	30 May 2019	Chihuahua	Juárez	Juárez	−106.487	31.739
MZ618802	Tropical Linage	♀	Environment	6 June 2019	Chihuahua	Juárez	Juárez	−106.487	31.739
MZ618803	Tropical Linage	♂	Dog	30 July 2019	Guanajuato	Guanajuato	Guanajuato	−101.263	21.019
MZ618804	Tropical Linage	♂	Dog	30 July 2019	Guanajuato	Guanajuato	Guanajuato	−101.263	21.019
MZ618805	Tropical Linage	♀	Dog	30 July 2019	Guanajuato	Guanajuato	Guanajuato	−101.263	21.019
MZ618806	Tropical Linage	♂	Dog	15 August 2019	Guanajuato	Guanajuato	Guanajuato	−101.263	21.019
MZ618807	Tropical Linage	♂	Environment	23 August 2019	Guanajuato	Dolores Hidalgo	Dolores Hidalgo	−101	21.100
MZ618808	Tropical Linage	♀	Dog	1 January 2019	Guerrero	Chilpancingo de los Bravo	Chilpancingo de los Bravo	−99.501	17.552
MZ618809	Tropical Linage	♀	Dog	1 January 2019	Guerrero	Chilpancingo de los Bravo	Chilpancingo de los Bravo	−99.501	17.552
MZ618810	Tropical Linage	♀	Dog	25 October 2019	Hidalgo	Chapulhuacan	Chapulhuacan	−98.904	21.155
MZ618811	Tropical Linage	♀	Dog	25 October 2019	Hidalgo	Chapulhuacan	Chapulhuacan	−98.904	21.155
MZ618812	Tropical Linage	♀	Dog	12 July 2019	Morelos	Tlaquiltenango	Tlaquiltenango	−99.160	18.629
MZ618813	Tropical Linage	♀	Dog	15 July 2019	Morelos	Tlaltizapán	Santa Rosa Treinta	−99.179	18.698
MZ618814	Tropical Linage	♀	Dog	12 July 2019	Morelos	Tlaquiltenango	Tlaquiltenango	−99.160	18.629
MZ618815	Tropical Linage	♀	Dog	15 July 2019	Morelos	Tlaltizapán	Santa Rosa Treinta	−99.179	18.698
MZ618816	Tropical Linage	♂	Dog	13 July 2019	Oaxaca	Sta. Lucía del Camino	Santa Lucía del Camino	−96.683	17.067
MZ618817	Tropical Linage	♂	Dog	13 June 2019	Oaxaca	Sta. Lucía del Camino	Santa Lucía del Camino	−96.683	17.067
MZ618818	Tropical Linage	♂	Dog	13 June 2019	Oaxaca	Sta. Lucía del Camino	Santa Lucía del Camino	−96.683	17.067
MZ618819	Tropical Linage	♀	Dog	18 July 2019	Oaxaca	Oaxaca de Juárez	Oaxaca de Juárez	−96.722	17.062
MZ618820	Tropical Linage	♀	Dog	1 August 2021	Oaxaca	Oaxaca de Juárez	Oaxaca de Juárez	−96.722	17.062
MZ618821	Tropical Linage	♀	Dog	6 July 2021	Puebla	Tehuacán	Tehuacán	−97.394	18.463
MZ618822	Tropical Linage	♀	Dog	6 July 2021	Puebla	Tehuacán	Tehuacán	−97.394	18.463
MZ618823	Tropical Linage	♀	Dog	19 September 2019	Puebla	Tehuacán	Tehuacán	−97.394	18.463
MZ618824	Tropical Linage	♀	Dog	19 September 2019	Puebla	Tehuacán	Tehuacán	−97.394	18.463
MZ618825	Tropical Linage	♂	Dog	24 September 2019	Puebla	Santiago Miahuatlán	Santiago Miahuatlán	−97.442	18.554
MZ618826	*R. sanguineus* s.s.	♀	Dog	4 June 2019	Sonora	Agua Prieta	Agua Prieta	−109.549	31.331
MZ618827	Tropical Linage	♂	Dog	4 June 2019	Sonora	Agua Prieta	Agua Prieta	−109.549	31.331
MZ618828	Tropical Linage	♀	Dog	24 June 2019	Sonora	Sta. Ana	Santa Ana	−111.121	30.541
MZ618829	Tropical Linage	♀	Dog	7 May 2019	Sonora	Fronteras	Esqueda	−109.590	32.721
MZ618830	Tropical Linage	♀	Dog	22 May 2019	Sonora	Magdalena	Magdalena de Kino	−110.969	30.625
MZ618831	Tropical Linage	♀	Dog	4 August 2019	Veracruz	Boca del Río	Boca del Río	−96.107	19.101
MZ618832	Tropical Linage	♀	Dog	8 February 2019	Yucatán	Mérida	Mérida	−89.62	20.970
MZ618833	Tropical Linage	♀	Dog	3 March 2019	Yucatán	Mérida	Mérida	−89.62	20.970
MZ618782	Tropical Linage	♀	Dog	12 March 2019	Yucatán	Mérida	Mérida	−89.62	20.970

## Data Availability

The data presented in this study are available in the Supplementary Materials.

## Supplementary Materials

The following are available online at https://www.mdpi.com/article/10.3390/pathogens10091118/s1. Supplementary File 1: A detailed list of localities; Supplementary File 2: Phylogenetic Inferences; Supplementary File 3: Genetic Analysis; Supplementary File 4: Geographic Distribution.
