# Supplementary figures and images for: Rhipicephalus sanguineus Complex in the Americas: Systematic, Genetic Diversity, and Geographic Insights
Source: Pathogens. 2021 Sep 1;10(9):1118. doi: 10.3390/pathogens10091118 (PMC8471700; doi:10.3390/pathogens10091118)

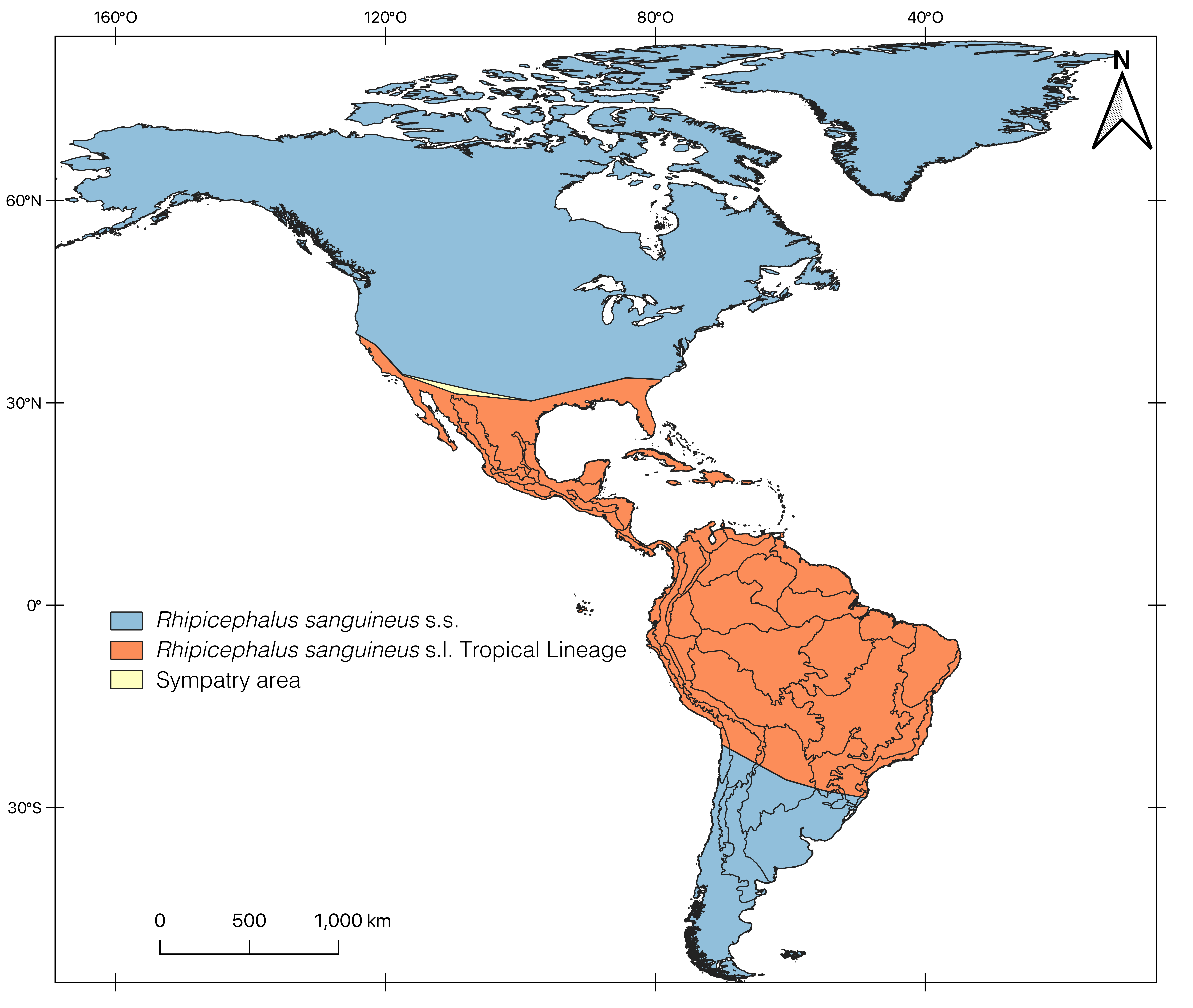

Supplement: Supplementary file 1 [file pathogens-10-01118-s001.zip › supplementary/Supplementary 4.0.tif]
