# Supplementary material for: Rhipicephalus sanguineus Complex in the Americas: Systematic, Genetic Diversity, and Geographic Insights
Source: Pathogens. 2021 Sep 1;10(9):1118. doi: 10.3390/pathogens10091118 (PMC8471700; doi:10.3390/pathogens10091118)
Supplement: Supplementary file 1 [file Supplementary 2.0.pdf › supplementary/Supplementary 2.0.pdf]

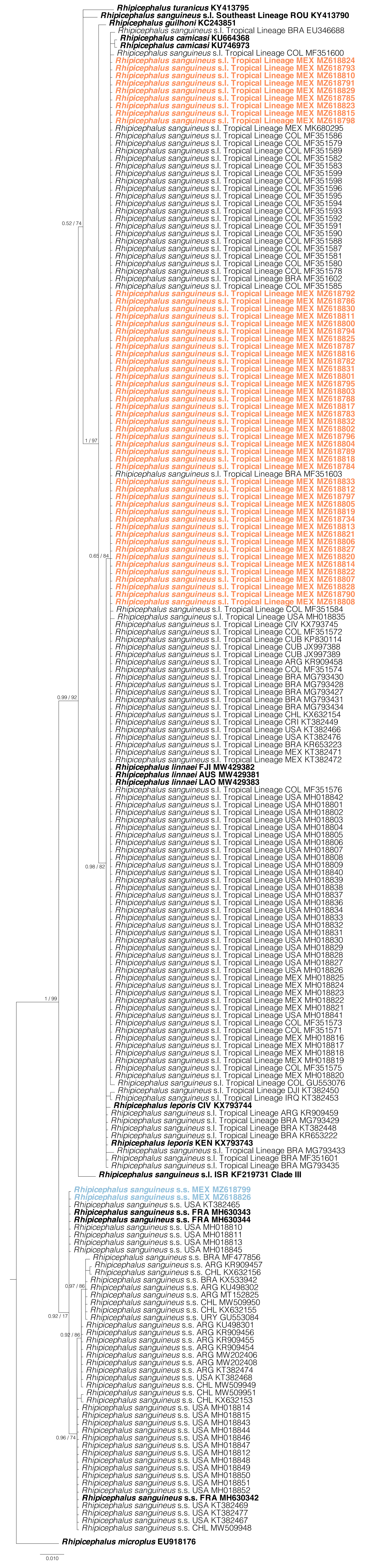
Rhipicephalus turanicus KY413795
Rhipicephalus sanguineus s.l. Southeast Lineage ROU KY413790
Rhipicephalus guilhoni KC243851
Rhipicephalus sanguineus s.l. Tropical Lineage BRA EU346688
Rhipicephalus camicasi KU664368
Rhipicephalus camicasi KU746973
Rhipicephalus sanguineus s.l. Tropical Lineage COL MF351600
Rhipicephalus sanguineus s.l. Tropical Lineage MEX MZ618824
Rhipicephalus sanguineus s.l. Tropical Lineage MEX MZ618793
Rhipicephalus sanguineus s.l. Tropical Lineage MEX MZ618810
Rhipicephalus sanguineus s.l. Tropical Lineage MEX MZ618791
Rhipicephalus sanguineus s.l. Tropical Lineage MEX MZ618829
Rhipicephalus sanguineus s.l. Tropical Lineage MEX MZ618785
Rhipicephalus sanguineus s.l. Tropical Lineage MEX MZ618823
Rhipicephalus sanguineus s.l. Tropical Lineage MEX MZ618815
Rhipicephalus sanguineus s.l. Tropical Lineage MEX MZ618798
Rhipicephalus sanguineus s.l. Tropical Lineage MEX MK680295
Rhipicephalus sanguineus s.l. Tropical Lineage COL MF351586
Rhipicephalus sanguineus s.l. Tropical Lineage COL MF351579
Rhipicephalus sanguineus s.l. Tropical Lineage COL MF351589
Rhipicephalus sanguineus s.l. Tropical Lineage COL MF351582
Rhipicephalus sanguineus s.l. Tropical Lineage COL MF351583
Rhipicephalus sanguineus s.l. Tropical Lineage COL MF351599
Rhipicephalus sanguineus s.l. Tropical Lineage COL MF351598
Rhipicephalus sanguineus s.l. Tropical Lineage COL MF351596
Rhipicephalus sanguineus s.l. Tropical Lineage COL MF351595
Rhipicephalus sanguineus s.l. Tropical Lineage COL MF351594
Rhipicephalus sanguineus s.l. Tropical Lineage COL MF351593
Rhipicephalus sanguineus s.l. Tropical Lineage COL MF351592
Rhipicephalus sanguineus s.l. Tropical Lineage COL MF351591
Rhipicephalus sanguineus s.l. Tropical Lineage COL MF351590
Rhipicephalus sanguineus s.l. Tropical Lineage COL MF351588
Rhipicephalus sanguineus s.l. Tropical Lineage COL MF351587
Rhipicephalus sanguineus s.l. Tropical Lineage COL MF351581
Rhipicephalus sanguineus s.l. Tropical Lineage COL MF351580
Rhipicephalus sanguineus s.l. Tropical Lineage COL MF351578
Rhipicephalus sanguineus s.l. Tropical Lineage BRA MF351602
Rhipicephalus sanguineus s.l. Tropical Lineage COL MF351585
Rhipicephalus sanguineus s.l. Tropical Lineage MEX MZ618792
Rhipicephalus sanguineus s.l. Tropical Lineage MEX MZ618786
Rhipicephalus sanguineus s.l. Tropical Lineage MEX MZ618830
Rhipicephalus sanguineus s.l. Tropical Lineage MEX MZ618811
Rhipicephalus sanguineus s.l. Tropical Lineage MEX MZ618800
Rhipicephalus sanguineus s.l. Tropical Lineage MEX MZ618794
Rhipicephalus sanguineus s.l. Tropical Lineage MEX MZ618825
Rhipicephalus sanguineus s.l. Tropical Lineage MEX MZ618787
Rhipicephalus sanguineus s.l. Tropical Lineage MEX MZ618816
Rhipicephalus sanguineus s.l. Tropical Lineage MEX MZ618782
Rhipicephalus sanguineus s.l. Tropical Lineage MEX MZ618831
Rhipicephalus sanguineus s.l. Tropical Lineage MEX MZ618801
Rhipicephalus sanguineus s.l. Tropical Lineage MEX MZ618795
Rhipicephalus sanguineus s.l. Tropical Lineage MEX MZ618803
Rhipicephalus sanguineus s.l. Tropical Lineage MEX MZ618788
Rhipicephalus sanguineus s.l. Tropical Lineage MEX MZ618817
Rhipicephalus sanguineus s.l. Tropical Lineage MEX MZ618783
Rhipicephalus sanguineus s.l. Tropical Lineage MEX MZ618832
Rhipicephalus sanguineus s.l. Tropical Lineage MEX MZ618802
Rhipicephalus sanguineus s.l. Tropical Lineage MEX MZ618796
Rhipicephalus sanguineus s.l. Tropical Lineage MEX MZ618804
Rhipicephalus sanguineus s.l. Tropical Lineage MEX MZ618789
Rhipicephalus sanguineus s.l. Tropical Lineage MEX MZ618818
Rhipicephalus sanguineus s.l. Tropical Lineage MEX MZ618784
Rhipicephalus sanguineus s.l. Tropical Lineage BRA MF351603
Rhipicephalus sanguineus s.l. Tropical Lineage MEX MZ618833
Rhipicephalus sanguineus s.l. Tropical Lineage MEX MZ618812
Rhipicephalus sanguineus s.l. Tropical Lineage MEX MZ618797
Rhipicephalus sanguineus s.l. Tropical Lineage MEX MZ618805
Rhipicephalus sanguineus s.l. Tropical Lineage MEX MZ618819
Rhipicephalus sanguineus s.l. Tropical Lineage MEX MZ618734
Rhipicephalus sanguineus s.l. Tropical Lineage MEX MZ618813
Rhipicephalus sanguineus s.l. Tropical Lineage MEX MZ618821
Rhipicephalus sanguineus s.l. Tropical Lineage MEX MZ618806
Rhipicephalus sanguineus s.l. Tropical Lineage MEX MZ618827
Rhipicephalus sanguineus s.l. Tropical Lineage MEX MZ618820
Rhipicephalus sanguineus s.l. Tropical Lineage MEX MZ618814
Rhipicephalus sanguineus s.l. Tropical Lineage MEX MZ618822
Rhipicephalus sanguineus s.l. Tropical Lineage MEX MZ618807
Rhipicephalus sanguineus s.l. Tropical Lineage MEX MZ618828
Rhipicephalus sanguineus s.l. Tropical Lineage MEX MZ618790
Rhipicephalus sanguineus s.l. Tropical Lineage MEX MZ618808
Rhipicephalus sanguineus s.l. Tropical Lineage COL MF351584
Rhipicephalus sanguineus s.l. Tropical Lineage USA MH018835
Rhipicephalus sanguineus s.l. Tropical Lineage CIV KX793745
Rhipicephalus sanguineus s.l. Tropical Lineage COL MF351572
Rhipicephalus sanguineus s.l. Tropical Lineage CUB KP830114
Rhipicephalus sanguineus s.l. Tropical Lineage CUB JX997388
Rhipicephalus sanguineus s.l. Tropical Lineage CUB JX997389
Rhipicephalus sanguineus s.l. Tropical Lineage ARG KR909458
Rhipicephalus sanguineus s.l. Tropical Lineage COL MF351574
Rhipicephalus sanguineus s.l. Tropical Lineage BRA MG793430
Rhipicephalus sanguineus s.l. Tropical Lineage BRA MG793428
Rhipicephalus sanguineus s.l. Tropical Lineage BRA MG793427
Rhipicephalus sanguineus s.l. Tropical Lineage BRA MG793431
Rhipicephalus sanguineus s.l. Tropical Lineage BRA MG793434
Rhipicephalus sanguineus s.l. Tropical Lineage CHL KX632154
Rhipicephalus sanguineus s.l. Tropical Lineage CRI KT382449
Rhipicephalus sanguineus s.l. Tropical Lineage USA KT382466
Rhipicephalus sanguineus s.l. Tropical Lineage USA KT382476
Rhipicephalus sanguineus s.l. Tropical Lineage BRA KR653223
Rhipicephalus sanguineus s.l. Tropical Lineage MEX KT382471
Rhipicephalus sanguineus s.l. Tropical Lineage MEX KT382472
Rhipicephalus linnaei FJI MW429382
Rhipicephalus linnaei AUS MW429381
Rhipicephalus linnaei LAO MW429383
Rhipicephalus sanguineus s.l. Tropical Lineage COL MF351576
Rhipicephalus sanguineus s.l. Tropical Lineage USA MH018842
Rhipicephalus sanguineus s.l. Tropical Lineage USA MH018801
Rhipicephalus sanguineus s.l. Tropical Lineage USA MH018802
Rhipicephalus sanguineus s.l. Tropical Lineage USA MH018803
Rhipicephalus sanguineus s.l. Tropical Lineage USA MH018804
Rhipicephalus sanguineus s.l. Tropical Lineage USA MH018805
Rhipicephalus sanguineus s.l. Tropical Lineage USA MH018806
Rhipicephalus sanguineus s.l. Tropical Lineage USA MH018807
Rhipicephalus sanguineus s.l. Tropical Lineage USA MH018808
Rhipicephalus sanguineus s.l. Tropical Lineage USA MH018809
Rhipicephalus sanguineus s.l. Tropical Lineage USA MH018840
Rhipicephalus sanguineus s.l. Tropical Lineage USA MH018839
Rhipicephalus sanguineus s.l. Tropical Lineage USA MH018838
Rhipicephalus sanguineus s.l. Tropical Lineage USA MH018837
Rhipicephalus sanguineus s.l. Tropical Lineage USA MH018836
Rhipicephalus sanguineus s.l. Tropical Lineage USA MH018834
Rhipicephalus sanguineus s.l. Tropical Lineage USA MH018833
Rhipicephalus sanguineus s.l. Tropical Lineage USA MH018832
Rhipicephalus sanguineus s.l. Tropical Lineage USA MH018831
Rhipicephalus sanguineus s.l. Tropical Lineage USA MH018830
Rhipicephalus sanguineus s.l. Tropical Lineage USA MH018829
Rhipicephalus sanguineus s.l. Tropical Lineage USA MH018828
Rhipicephalus sanguineus s.l. Tropical Lineage USA MH018827
Rhipicephalus sanguineus s.l. Tropical Lineage USA MH018826
Rhipicephalus sanguineus s.l. Tropical Lineage MEX MH018825
Rhipicephalus sanguineus s.l. Tropical Lineage MEX MH018824
Rhipicephalus sanguineus s.l. Tropical Lineage MEX MH018823
Rhipicephalus sanguineus s.l. Tropical Lineage MEX MH018822
Rhipicephalus sanguineus s.l. Tropical Lineage MEX MH018821
Rhipicephalus sanguineus s.l. Tropical Lineage USA MH018841
Rhipicephalus sanguineus s.l. Tropical Lineage COL MF351573
Rhipicephalus sanguineus s.l. Tropical Lineage COL MF351571
Rhipicephalus sanguineus s.l. Tropical Lineage MEX MH018816
Rhipicephalus sanguineus s.l. Tropical Lineage MEX MH018817
Rhipicephalus sanguineus s.l. Tropical Lineage MEX MH018818
Rhipicephalus sanguineus s.l. Tropical Lineage MEX MH018819
Rhipicephalus sanguineus s.l. Tropical Lineage COL MF351575
Rhipicephalus sanguineus s.l. Tropical Lineage MEX MH018820
Rhipicephalus sanguineus s.l. Tropical Lineage COL GU553076
Rhipicephalus sanguineus s.l. Tropical Lineage DJI KT382450
Rhipicephalus sanguineus s.l. Tropical Lineage IRQ KT382453
Rhipicephalus leporis CIV KX793744
Rhipicephalus sanguineus s.l. Tropical Lineage ARG KR909459
Rhipicephalus sanguineus s.l. Tropical Lineage BRA MG793429
Rhipicephalus sanguineus s.l. Tropical Lineage BRA KT382448
Rhipicephalus sanguineus s.l. Tropical Lineage BRA KR653222
Rhipicephalus leporis KEN KX793743
Rhipicephalus sanguineus s.l. Tropical Lineage BRA MG793433
Rhipicephalus sanguineus s.l. Tropical Lineage BRA MF351601
Rhipicephalus sanguineus s.l. Tropical Lineage BRA MG793435
Rhipicephalus sanguineus s.l. ISR KF219731 Clade III
Rhipicephalus sanguineus s.s. MEX MZ618799
Rhipicephalus sanguineus s.s. MEX MZ618826
Rhipicephalus sanguineus s.s. USA KT382465
Rhipicephalus sanguineus s.s. FRA MH630343
Rhipicephalus sanguineus s.s. FRA MH630344
Rhipicephalus sanguineus s.s. USA MH018810
Rhipicephalus sanguineus s.s. USA MH018811
Rhipicephalus sanguineus s.s. USA MH018813
Rhipicephalus sanguineus s.s. USA MH018845
Rhipicephalus sanguineus s.s. BRA MF477856
Rhipicephalus sanguineus s.s. ARG KR909457
Rhipicephalus sanguineus s.s. CHL KX632156
Rhipicephalus sanguineus s.s. BRA KX533942
Rhipicephalus sanguineus s.s. ARG KU498302
Rhipicephalus sanguineus s.s. ARG MT152825
Rhipicephalus sanguineus s.s. CHL MW509950
Rhipicephalus sanguineus s.s. CHL KX632155
Rhipicephalus sanguineus s.s. URY GU553084
Rhipicephalus sanguineus s.s. ARG KU498301
Rhipicephalus sanguineus s.s. ARG KR909456
Rhipicephalus sanguineus s.s. ARG KR909455
Rhipicephalus sanguineus s.s. ARG KR909454
Rhipicephalus sanguineus s.s. ARG MW202406
Rhipicephalus sanguineus s.s. ARG MW202408
Rhipicephalus sanguineus s.s. ARG KT382474
Rhipicephalus sanguineus s.s. USA KT382468
Rhipicephalus sanguineus s.s. CHL MW509949
Rhipicephalus sanguineus s.s. CHL MW509951
Rhipicephalus sanguineus s.s. CHL KX632153
Rhipicephalus sanguineus s.s. USA MH018814
Rhipicephalus sanguineus s.s. USA MH018815
Rhipicephalus sanguineus s.s. USA MH018843
Rhipicephalus sanguineus s.s. USA MH018844
Rhipicephalus sanguineus s.s. USA MH018846
Rhipicephalus sanguineus s.s. USA MH018847
Rhipicephalus sanguineus s.s. USA MH018812
Rhipicephalus sanguineus s.s. USA MH018848
Rhipicephalus sanguineus s.s. USA MH018849
Rhipicephalus sanguineus s.s. USA MH018850
Rhipicephalus sanguineus s.s. USA MH018851
Rhipicephalus sanguineus s.s. USA MH018852
Rhipicephalus sanguineus s.s. FRA MH630342
Rhipicephalus sanguineus s.s. USA KT382469
Rhipicephalus sanguineus s.s. USA KT382477
Rhipicephalus sanguineus s.s. USA KT382467
Rhipicephalus sanguineus s.s. CHL MW509948
Rhipicephalus microplus EU918176
0.52 / 74
1 / 97
0.65 / 84
0.99 / 92
0.98 / 82
1 / 99
0.97 / 86
0.92 / 17
0.92 / 86
0.96 / 74
0.010
